# Correction: Santori et al. Impact of *Salmonella enteritidis* Infection and Mechanical Stress on Antimicrobial Peptide Expression in *Hermetia illucens*. *Insects* 2025, *16*, 692

**DOI:** 10.3390/insects16111174

**Published:** 2025-11-18

**Authors:** Davide Santori, Anna Maria Fausto, Alessio Gelli, Anna Rita Pifferi, Samuele Dottarelli, Sofia Cucci, Francesca Di Donato, Goffredo Grifoni, Erminia Sezzi

**Affiliations:** 1 Istituto Zooprofilattico Sperimentale del Lazio e della Toscana “M. Aleandri”, 00149 Roma, RM, Italy; alessio.gelli@izslt.it (A.G.); annarita.pifferi@izslt.it (A.R.P.); samuele.dottarelli@izslt.it (S.D.); sofia.cucci-esterno@izslt.it (S.C.); francesca.didonato@izslt.it (F.D.D.); goffredo.grifoni@izslt.it (G.G.); erminia.sezzi@izslt.it (E.S.); 2 Department for Innovation in Biological, Agro-Food and Forest Systems (DIBAF), University of Tuscia, Via San Camillo De Lellis snc, 01100 Viterbo, VT, Italy; fausto@unitus.it

## Missing Acknowledgements

In the original publication [1], acknowledgements were not included. We want to thank Marco Gerdol of the University of Trieste for his invaluable contribution in developing the specific primers utilized in this research. 

The authors state that the scientific conclusions are unaffected. This correction was approved by the Academic Editor. The original publication has also been updated.
